# Localized Depletion of Seminal HDL-C Despite Preserved Systemic Lipid Profiles in Men with Impaired Semen Parameters: A Prospective Cross-Sectional Study

**DOI:** 10.3390/biom16060820

**Published:** 2026-06-01

**Authors:** Merve Huner Yigit, Ertugrul Yigit, Mehtap Atak, Hakki Uzun

**Affiliations:** 1 Department of Medical Biochemistry, Faculty of Medicine, Recep Tayyip Erdoğan University, Rize 53100, Türkiye; mehtap.atak@erdogan.edu.tr; 2 Department of Medical Biochemistry, Faculty of Medicine, Karadeniz Technical University, Trabzon 61080, Türkiye; ertugrulyigit@ktu.edu.tr; 3 Department of Urology, Faculty of Medicine, Recep Tayyip Erdoğan University, Rize 53100, Türkiye; hakki.uzun@erdogan.edu.tr

**Keywords:** oligoasthenoteratozoospermia, seminal HDL cholesterol, HDL subfractions, male infertility, sperm motility

## Abstract

Background: Lipid homeostasis is essential for sperm membrane integrity, capacitation, and fertilizing competence. However, whether lipid alterations associated with oligoasthenoteratozoospermia (OAT) reflect systemic dyslipidemia or a disturbance localized to the seminal compartment remains unclear. This study investigated serum high-density lipoprotein (HDL) subfractions and seminal lipid concentrations in men with OAT. Methods: In this prospective cross-sectional study, 99 men were included: 49 men clinically classified as having OAT and 50 men with normozoospermia. Conventional semen analysis was performed according to the WHO 2021 manual. Serum HDL subfractions were analyzed using the Lipoprint HDL system, which classifies HDL into 10 subfractions and 3 major groups (large, intermediate, and small HDL). Seminal plasma total cholesterol and high-density lipoprotein cholesterol (HDL-C) were measured using enzymatic colorimetric and fluorometric assays, respectively. Correlations between lipid parameters and semen quality indices were assessed using Spearman’s rank analysis. Results: Baseline demographic and systemic metabolic characteristics were comparable between groups. Men with OAT had significantly higher FSH and estradiol levels and markedly impaired semen parameters, including sperm concentration, total sperm count, total motile sperm count, motility, and morphology. No significant differences were observed in serum HDL subfractions 1–10 or in large, intermediate, and small HDL concentrations between groups. In contrast, seminal total cholesterol was significantly lower in the OAT group (*p* = 0.048), and seminal HDL-C was markedly reduced (*p* < 0.001). Seminal HDL-C showed weak-to-moderate positive correlations with sperm concentration (ρ = 0.407), rapid progressive motility (ρ = 0.417), slow progressive motility (ρ = 0.418), total motile sperm count (ρ = 0.379), and normal morphology (ρ = 0.344) (all *p* < 0.001). Conclusions: OAT is characterized by a compartmentalized lipid alteration marked by preserved systemic HDL subfraction profiles but depleted seminal HDL-C. These findings suggest that local seminal lipid homeostasis may be more closely related to sperm quality than circulating HDL-related measures and support seminal HDL-C as a candidate local metabolic indicator in male infertility.

## 1. Introduction

Infertility is a major global health problem, affecting approximately 15% of couples attempting conception through regular unprotected sexual intercourse, with a male factor contributing to nearly half of all cases [1]. Although semen analysis remains the cornerstone of male infertility evaluation, conventional parameters may not fully capture the functional and metabolic disturbances underlying impaired fertilizing capacity. In this context, men presenting with combined abnormalities in sperm concentration, motility, and morphology, referred to here as an oligoasthenoteratozoospermia (OAT) phenotype, represent a clinically relevant subgroup for studying severe composite semen impairment. While the WHO 2021 manual emphasizes decision limits rather than rigid syndromic labels [2], such classifications remain useful for descriptive and comparative research. Male infertility is etiologically heterogeneous, encompassing pre-testicular, testicular, and post-testicular causes, with idiopathic forms accounting for a substantial proportion of cases [3,4]. Accordingly, the biological basis of male infertility may reside not only in reduced sperm number, but also in disturbances of sperm membrane organization and its surrounding metabolic milieu.

The sperm plasma membrane has a highly specialized lipid architecture that is essential for membrane stability and fertilization competence. It is enriched in phospholipids, polyunsaturated fatty acids, and cholesterol, which together contribute to its structural and functional properties [5]. The asymmetric distribution of phospholipids across the sperm plasma membrane bilayer is dynamically regulated and plays a critical role in signaling events during capacitation and the acrosome reaction [6]. Cholesterol homeostasis is particularly important during capacitation, during which controlled cholesterol efflux promotes membrane remodeling, redistribution of membrane microdomains, and preparation for the acrosome reaction [7,8]. In this context, bicarbonate-dependent membrane remodeling has been shown to facilitate albumin-mediated cholesterol efflux and lateral redistribution of cholesterol within the sperm head plasma membrane, consistent with apical membrane raft formation [9]. Disturbances in this lipid balance may therefore compromise sperm function.

High-density lipoprotein (HDL), the principal lipoprotein involved in reverse cholesterol transport, is a plausible mediator of this process. Beyond its systemic role in cholesterol trafficking, HDL has also been identified in reproductive fluids and implicated in the removal of cholesterol from sperm membranes, thereby facilitating fertilization-related functions. This mechanism has been demonstrated most directly in animal models, including ram spermatozoa, in which HDL promotes cholesterol efflux and hyperactivated motility [10]. These observations support the concept that HDL may participate in a localized cholesterol-cycling system within the seminal compartment rather than merely reflecting circulating lipid status.

Recent human data further suggest that systemic and seminal lipid environments should not be considered interchangeable. Seminal plasma constitutes a complex biochemical milieu derived from the testis, epididymis, seminal vesicles, and prostate, and its composition reflects both local secretory function and systemic influences [11]. Płaczkowska et al. simultaneously evaluated blood serum and seminal plasma biochemistry and showed that biochemical associations with semen quality may differ by the biological compartment examined [12]. This distinction is biologically plausible because the blood–testis barrier (BTB) creates a highly specialized microenvironment for germ cell development and limits direct equilibration between circulating and seminal components [13]. The BTB, formed by adjacent Sertoli cells near the basement membrane, separates the seminiferous epithelium into basal and adluminal compartments and provides a specialized microenvironment for germ cell development [14]. Therefore, inconsistent findings in the literature on lipids and semen quality may partly reflect an overreliance on systemic measurements, whereas functionally relevant lipid disturbances may be localized within the reproductive tract.

Another limitation of the existing literature is the tendency to treat HDL as a single uniform entity. HDL is heterogeneous and can be resolved into distinct subfractions with potentially different biological properties. The large HDL subfractions are generally considered to possess greater anti-inflammatory and cholesterol efflux capacity than small, dense HDL particles, underscoring the functional heterogeneity of HDL subclasses [15]. Analytical systems such as Lipoprint classify HDL into large, intermediate, and small subfractions, enabling a more detailed characterization than total HDL Cholesterol (HDL-C) alone. At the same time, targeted assessment of seminal lipids may provide information not captured by serum measurements alone. Against this background, the present study was designed to evaluate systemic and local lipid compartments simultaneously in the same individuals. Specifically, we analyzed serum HDL subfractions using the Lipoprint^®^ system and quantified seminal plasma total cholesterol and HDL-C to determine whether men with an OAT phenotype exhibit evidence of compartmentalized lipid dysregulation associated with impaired sperm quality.

## 2. Materials and Methods

### 2.1. Study Design, Setting, and Ethical Approval

This prospective cross-sectional study was conducted at the Andrology Outpatient Clinic of Recep Tayyip Erdoğan University Training and Research Hospital. Before participant recruitment, sample size estimation was performed using G*Power (version 3.1.9.7, Heinrich-Heine-Universität Düsseldorf, Germany). Assuming a two-tailed alpha level of 0.05, a statistical power of 80%, and a medium effect size of 0.60 for between-group comparisons, the minimum required sample size was calculated as 88 participants. To strengthen the statistical reliability of the study, 99 participants were ultimately included, comprising 49 men with impaired semen parameters and 50 men with normozoospermia. The study protocol was reviewed and approved by the Non-Interventional Local Ethics Committee of Recep Tayyip Erdoğan University on 26 September 2024 (Approval No. 2024/254). All procedures were carried out in accordance with the principles of the Declaration of Helsinki, and written informed consent was obtained from all participants before enrolment [16].

### 2.2. Participant Selection and Eligibility Criteria

Group allocation was based on clinical evaluation and semen analysis findings. Semen parameters were interpreted according to the lower decision limits reported in the World Health Organization (WHO) Laboratory Manual for the Examination and Processing of Human Semen, 6th edition [2]. For descriptive and comparative purposes in this study, men were classified as having an OAT phenotype when they concurrently exhibited reduced sperm concentration (<16 × 10^6^/mL), reduced progressive motility (rapid + slow progressive motility <30%), and reduced normal morphology (<4% according to Kruger’s strict criteria). This classification was applied strictly; men who met only one or two of the three criteria were not assigned to the OAT group and were excluded from the study to ensure phenotypic homogeneity. Men in the control group had normozoospermic semen parameters according to the same manual. Control participants were recruited from men attending the same clinic for routine fertility evaluation as part of couple-based infertility workups, whose partners were subsequently identified as having an underlying cause for infertility. This approach was adopted to minimize selection bias and ensure comparable demographic backgrounds between groups. To minimize potential confounding, a broad set of exclusion criteria was applied. Men were excluded if they had clinical varicocele, cryptorchidism, prior inguinal or scrotal surgery, active genitourinary infection, chronic systemic disease, endocrine dysfunction, known genetic causes of male infertility, or a history of chemotherapy or radiotherapy. Participants were also excluded if they had used antioxidants, vitamin preparations, hormonal agents, corticosteroids, anabolic steroids, or lipid-lowering drugs within the preceding 3 months, or had experienced a recent febrile illness that might adversely affect spermatogenesis. These exclusion criteria were selected to reduce bias from conditions and exposures known to affect semen quality, spermatogenesis, or male infertility evaluation [2,17].

### 2.3. Semen Collection, Conventional Semen Analysis, and Morphological Evaluation

Semen samples were obtained by masturbation into sterile containers after 3 days of sexual abstinence. Following collection, samples were allowed to liquefy at 37 °C for 30 min before analysis. Conventional semen analysis was performed manually in the Andrology Laboratory using standard procedures, and semen parameters were interpreted with reference to the WHO Laboratory Manual for the Examination and Processing of Human Semen, 6th edition [2]. Semen volume, pH, viscosity, liquefaction, sperm concentration, and motility were evaluated as part of the routine laboratory workflow, and sperm motility was classified as rapid progressive, slow progressive, non-progressive, and immotile, consistent with the four-category motility system adopted in the WHO 6th edition [2,18]. For morphological assessment, semen smears were prepared and evaluated according to Kruger’s strict criteria. Only spermatozoa fulfilling strict morphologic requirements for the head, midpiece, and tail were classified as normal [2,19].

### 2.4. Blood Sampling and Routine Biochemical and Hormonal Measurements

After an 8–12 h overnight fast, venous blood samples were collected from all participants, and serum was separated by centrifugation. Routine biochemical parameters, including fasting glucose, total cholesterol, triglycerides, HDL-C, and Low-density lipoprotein Cholesterol (LDL-C), as well as reproductive hormones, including follicle-stimulating hormone (FSH), luteinizing hormone (LH), total testosterone (TT), and estradiol (E2), were measured using a Beckman Coulter AU5800 fully automated chemistry analyzer (Beckman Coulter Inc., Brea, CA, USA) with original manufacturer reagents and standard laboratory procedures.

### 2.5. Serum HDL Subfraction Analysis

Detailed analysis of serum HDL subfractions was performed using the Lipoprint HDL System (Quantimetrix Corp., Redondo Beach, CA, USA), a non-denaturing linear polyacrylamide gel electrophoresis-based method that separates and quantifies up to 10 HDL subfractions in serum or plasma. According to the manufacturer’s classification, HDL subfractions were further grouped into Large HDL (subfractions 1–3), Intermediate HDL (subfractions 4–7), and Small HDL (subfractions 8–10). All analyses were carried out in accordance with the manufacturer’s instructions.

### 2.6. Seminal Plasma Preparation and Lipid Measurements

After completion of routine semen analysis, seminal plasma was obtained by centrifuging semen samples at 3000× *g* for 10 min. The cell-free supernatant was carefully collected without sperm lysis or sonication, and stored at −80 °C until biochemical analysis. Seminal total cholesterol was measured using the Abcam Total Cholesterol & Cholesteryl Ester Colorimetric Assay Kit (ab282928; Abcam Ltd., Cambridge, UK) on a SpectraMax Paradigm multi-mode microplate reader (Molecular Devices, LLC, San Jose, CA, USA) by an enzymatic colorimetric method, according to the manufacturer’s instructions. Seminal HDL-C was measured using the Abcam Cholesterol Assay Kit—HDL (ab65390; Abcam Ltd., Cambridge, UK) on the same SpectraMax Paradigm platform using the fluorometric assay format, in accordance with the manufacturer’s protocol. All seminal lipid measurements were performed in duplicate, and both intra-assay and inter-assay coefficients of variation were below 10%, confirming the analytical reliability of the assays in the seminal plasma matrix. Thus, systemic HDL was characterized at the subfraction level, whereas seminal lipid assessment was limited to quantitative measurements of total cholesterol and HDL-C.

### 2.7. Statistical Analysis

Statistical analyses were performed using OriginPro 2025 (OriginLab Corp., Northampton, MA, USA). Normality of data distribution was assessed using the Shapiro–Wilk test. Because most continuous variables did not follow a normal distribution, they were summarized as median values with interquartile ranges (Q1–Q3). Categorical variables were expressed as a number (n) and percentage (%). Between-group comparisons for continuous variables were performed using the Mann–Whitney U test. Categorical variables, including smoking and alcohol status, where applicable, were compared using the Pearson chi-square test. Associations between lipid parameters and semen quality indices were initially assessed using Spearman’s rank correlation analysis. To further examine whether these associations were influenced by baseline endocrine differences, age, and smoking status between groups, additional covariate-adjusted partial correlation analyses were performed with serum FSH, E2, age, and smoking status included as control variables. All statistical tests were two-tailed, and a *p*-value < 0.05 was considered statistically significant.

## 3. Results

### 3.1. Baseline Demographic and Clinical Characteristics

The study cohort consisted of 99 men, including 49 men with OAT and 50 men with normozoospermia. Baseline demographic and systemic metabolic characteristics are summarized in Table 1. Age, height, weight, body mass index, smoking status, and alcohol consumption did not differ significantly between the groups (all *p* > 0.05). Likewise, fasting glucose and routine serum lipid parameters, including total cholesterol, triglycerides, HDL cholesterol, LDL cholesterol, and non-HDL cholesterol, were similar in men with OAT and men with normozoospermia (all *p* > 0.05). Overall, these findings indicate that the two groups were comparable in terms of baseline anthropometric features and systemic metabolic profile.

### 3.2. Endocrine Profile and Semen Parameters

Reproductive hormone levels and conventional semen parameters are summarized in Table 2. Although LH and total testosterone did not differ significantly between groups (both *p* > 0.05), serum FSH and estradiol levels were significantly higher in the OAT group than in controls (*p* = 0.008 and *p* = 0.015, respectively), indicating differences in the endocrine profile between groups. Consistent with the clinical phenotype, the OAT group exhibited marked impairment across conventional semen variables. Sperm concentration, total sperm count, and total motile sperm count were all significantly lower in men with OAT (all *p* < 0.001). Rapid progressive, slow progressive, and non-progressive motility percentages were also reduced, whereas the proportion of immotile sperm was significantly increased (all *p* < 0.001). In addition, normal morphology was markedly impaired in the OAT group, with a median value of 0% compared with 6% in men with normozoospermia (*p* < 0.001).

### 3.3. Serum HDL Subfractions and Seminal Lipid Profile

**Figure 1:**
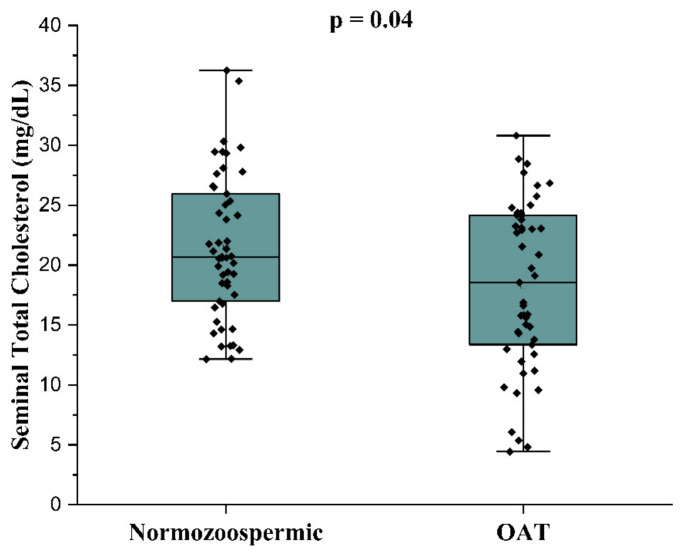
Seminal total cholesterol concentrations in men with normozoospermia (*n* = 50) and men with oligoasthenoteratozoospermia (OAT; *n* = 49). Box plots display the median (horizontal line), interquartile range (box), and 1.5× IQR (whiskers). Individual data points are shown as overlaid dots. Between-group comparison was performed using the Mann–Whitney U test (*p* = 0.048).

**Figure 2:**
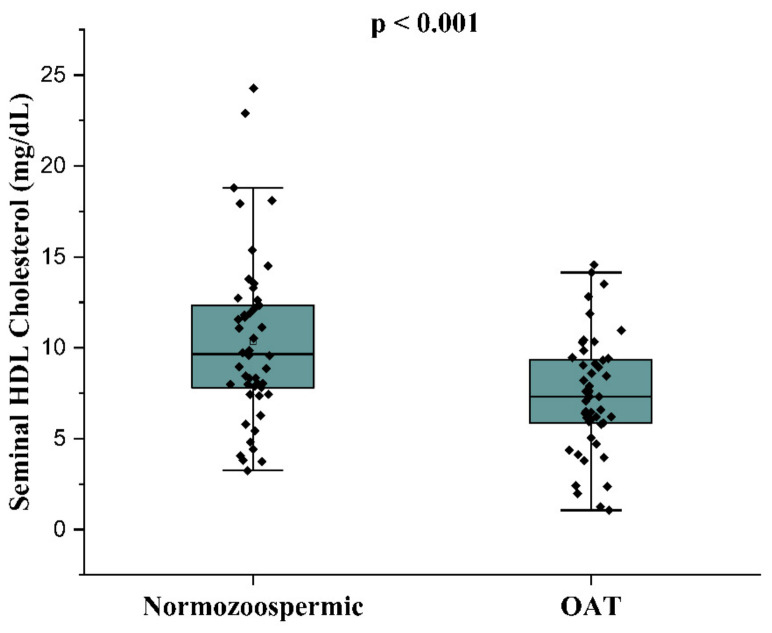
Seminal HDL cholesterol concentrations in men with normozoospermia (*n* = 50) and men with oligoasthenoteratozoospermia (OAT; *n* = 49). Box plots display the median (horizontal line), interquartile range (box), and 1.5× IQR (whiskers). Individual data points are shown as overlaid dots. Between-group comparison was performed using the Mann–Whitney U test (*p* < 0.001).

To explore lipid-related alterations associated with OAT, both systemic serum HDL fractions and local seminal lipid concentrations were compared between men with normozoospermia and men with OAT. As shown in Table 3, no significant between-group differences were observed in serum HDL subfractions 1–10 or in the absolute concentrations of Large HDL, Intermediate HDL, and Small HDL (all *p* > 0.05). In contrast, the seminal lipid profile differed significantly between the groups. Seminal total cholesterol levels were significantly lower in the OAT group than in controls (*p* = 0.048; Figure 1). A more pronounced difference was observed for seminal HDL cholesterol, which was markedly reduced in men with oligoasthenoteratozoospermia compared to men with normozoospermia (*p* < 0.001; Figure 2). Furthermore, when seminal lipid levels were expressed as total content per ejaculate, seminal HDL-C remained significantly lower in the OAT group (*p* = 0.010), whereas total cholesterol content per ejaculate did not differ significantly between groups (*p* = 0.194). Taken together, these findings support the presence of a compartmentalized lipid alteration in OAT, characterized by preserved systemic HDL subfraction distribution but reduced seminal HDL-related content.

### 3.4. Correlations Between Seminal Lipid Parameters and Sperm Quality

**Figure 3:**
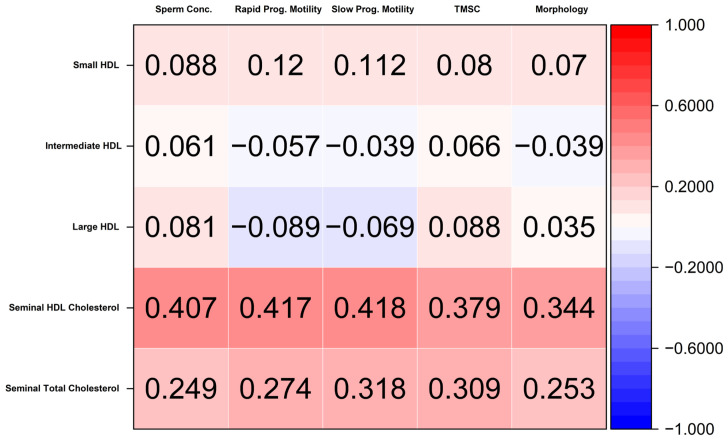
Heatmap of Spearman’s correlation coefficients between lipid profiles, hormones, and semen parameters. Red and blue colors represent positive and negative correlation coefficients, respectively. The intensity of the color indicates the strength of the relationship. HDL: High-Density Lipoprotein; TMSC: Total Motile Sperm Count.

Spearman’s correlation analysis across the full cohort (*n* = 99) showed that seminal HDL-C was significantly associated with key semen quality indices. Specifically, seminal HDL-C demonstrated weak-to-moderate positive correlations with sperm concentration (ρ = 0.407, *p* < 0.001), rapid progressive motility (ρ = 0.417, *p* < 0.001), slow progressive motility (ρ = 0.418, *p* < 0.001), total motile sperm count (ρ = 0.379, *p* < 0.001), and normal morphology (ρ = 0.344, *p* < 0.001). Seminal total cholesterol likewise showed significant positive correlations with sperm concentration (ρ = 0.249, *p* = 0.013), rapid progressive motility (ρ = 0.274, *p* = 0.007), slow progressive motility (ρ = 0.318, *p* = 0.002), total motile sperm count (ρ = 0.309, *p* = 0.005), and normal morphology (ρ = 0.253, *p* = 0.011). In contrast, no meaningful associations were observed between serum HDL subfractions, including Large HDL, Intermediate HDL, and Small HDL, and conventional semen parameters (all *p* > 0.05) (Figure 3). Collectively, these findings suggest that local seminal lipid homeostasis may be more closely related to sperm quality than circulating HDL-related measures.

### 3.5. Partial Correlation Analysis

To further examine whether the observed associations between seminal lipid parameters and semen quality were influenced by baseline differences between groups, partial correlation analyses were performed with serum FSH, E2, age, and smoking status included as covariates. After adjustment, seminal HDL-C remained positively correlated with rapid progressive motility (r = 0.409, *p* < 0.01), slow progressive motility (r = 0.379, *p* < 0.01), and normal morphology (r = 0.346, *p* < 0.01). Similarly, seminal total cholesterol remained positively correlated with rapid progressive motility (r = 0.333, *p* < 0.01), slow progressive motility (r = 0.381, *p* < 0.01), and normal morphology (r = 0.262, *p* = 0.01) (Table 4). Overall, these adjusted analyses indicate that the associations between reduced seminal lipid levels and impaired semen quality persist after accounting for differences in serum FSH, estradiol, age, and smoking status.

## 4. Discussion

The present study supports a compartment-specific pattern of lipid alteration in men with an OAT phenotype. The principal finding was that, despite preserved serum HDL profiles across all ten subfractions, including large, intermediate, and small HDL, men with OAT exhibited significantly reduced seminal HDL-C together with lower seminal total cholesterol. In addition, seminal HDL-C showed statistically significant weak-to-moderate positive correlations with sperm concentration, rapid progressive motility, and normal morphology. After adjustment for serum FSH, estradiol, age, and smoking status, the associations with rapid progressive motility, slow progressive motility, and normal morphology remained significant, suggesting that they were not fully explained by baseline endocrine differences between groups. Taken together, these findings indicate that conventional systemic lipid measurements may not adequately reflect the metabolic environment most relevant to sperm quality and that local lipid homeostasis within the seminal compartment may be more closely linked to semen quality than circulating HDL-related measures. This interpretation may also help explain the heterogeneous findings in the literature on lipids and semen quality, with previous studies reporting significant but complex associations across serum and seminal compartments rather than a single consistent systemic pattern [12,20]. In line with this perspective, Bi et al. showed in a large clinic-based cohort that hyperlipidemia was not accompanied by significant differences in conventional semen parameters, whereas serum testosterone levels were lower in men with hyperlipidemia and inversely related to triglyceride levels, further supporting the view that systemic lipid disturbances may be more readily reflected in endocrine markers than in routine semen indices [21].

This apparent dissociation between systemic and seminal lipid findings is biologically plausible in light of the BTB. The BTB establishes a highly specialized microenvironment for spermatogenesis and restricts direct equilibration between circulating and seminiferous compartments [13]. Within this regulated setting, Sertoli cells are central to local cholesterol handling. They take up cholesterol through receptors such as scavenger receptor class B type I (SR-BI) and regulate cholesterol trafficking through transporters including ATP-binding cassette transporter A1 (ABCA1), thereby contributing to a controlled local cholesterol pool required for germ-cell support and spermatogenesis. Notably, disruption of ABCA1-mediated cholesterol efflux in Sertoli cells results in intracellular cholesterol ester accumulation and impaired spermatogenesis in experimental models, underscoring the physiological necessity of tightly regulated local lipid homeostasis [22]. Furthermore, de Neergaard et al. demonstrated in a cross-sectional cohort study that seminal plasma total cholesterol was positively associated with sperm concentration, total sperm count, motility, and morphology, while showing no correlation with serum cholesterol levels—a finding that strongly aligns with the compartmentalized pattern reported in the present study [23]. Against this background, our findings support the possibility that the OAT phenotype is associated less with a generalized defect in systemic HDL distribution and more with a disturbance of local lipid trafficking or compartment-specific cholesterol regulation within the male reproductive tract.

From a functional perspective, the relevance of seminal HDL depletion may be most evident during sperm capacitation. Cholesterol efflux from the sperm plasma membrane is a prerequisite for membrane remodeling, increased membrane fluidity, lipid raft redistribution, and activation of downstream signaling events necessary for fertilization competence [8]. Recent work by Serafini and O’Flaherty further demonstrated that dysregulation of cholesterol and sphingolipid homeostasis impairs capacitation-associated tyrosine phosphorylation and PI3K signaling in human spermatozoa, providing mechanistic evidence that seminal lipid imbalances may compromise capacitation at the molecular level [24]. Experimental studies have further shown that HDL-containing extracellular environments can serve as cholesterol acceptors and facilitate sperm functional maturation, including hyperactivation-related changes [10,25]. In this context, reduced seminal HDL-C may indicate a less favorable extracellular milieu for cholesterol efflux, potentially contributing to impaired membrane dynamics and reduced sperm function. The observed associations of seminal HDL-C with sperm concentration, rapid progressive motility, and morphology are consistent with this biological framework, although the cross-sectional nature of the present study does not permit causal inference.

Beyond its potential role in cholesterol efflux during capacitation, reduced seminal HDL-C may also reflect a less favorable antioxidant milieu within the seminal compartment. Paraoxonase family members, particularly HDL-associated antioxidant enzymes, have been linked to semen quality and oxidative balance. Verit et al. reported that seminal PON-1 activity was lower in subfertile men with abnormal semen parameters and was positively correlated with sperm concentration, motility, and morphology [26]. More recently, Janati et al. observed that higher seminal paraoxonase-3 concentrations were associated with higher progressive motility and lower DNA fragmentation [27], whereas Dhillon et al. showed that oligospermic men had lower seminal antioxidant capacity together with reduced sirtuin 1 and sirtuin 3 levels, accompanied by higher malondialdehyde levels, greater DNA fragmentation, and shorter sperm telomere length [28]. Consistent with these observations, Wang et al. highlighted that excessive reactive oxygen species generation within the seminal compartment promotes lipid peroxidation of sperm membranes, DNA strand breaks, and protein oxidation, collectively impairing motility, morphology, and fertilization competence [29]. This oxidative vulnerability of spermatozoa is compounded by the fact that their lipid-rich plasma membranes and limited post-meiotic DNA repair capacity render them particularly susceptible to redox damage, as recently emphasized by Kaltsas et al. [30]. In this context, the marked reduction in seminal HDL-C observed in our study may be relevant not only to membrane cholesterol dynamics but also to diminished protection against oxidative stress within the seminal microenvironment. Although oxidative stress markers, paraoxonase activity, and DNA fragmentation were not directly assessed in the present study, these pathways provide a biologically plausible complementary framework linking compartment-specific lipid depletion to impaired semen quality.

Methodologically, one of the strengths of this study is the simultaneous evaluation of systemic HDL subfractions and local seminal lipid concentrations in the same individuals. The complete absence of differences across serum HDL subfractions argues against a major systemic HDL abnormality in the OAT phenotype, whereas the significant reduction in seminal HDL-C points to a compartment-specific disturbance. This distinction is important because male infertility evaluations often rely predominantly on systemic biochemical parameters, which may not capture biologically relevant alterations within the reproductive tract. In this regard, Moustakli et al. emphasized in a narrative review that seminal plasma represents a particularly rich and non-invasive source of candidate biomarkers, and that its biochemical profiling may provide clinically meaningful information that complements conventional semen analysis in the evaluation of male infertility [31]. Our findings, therefore, support the concept that seminal biochemical profiling may provide complementary information beyond conventional serum lipid testing in mechanistic studies of male infertility.

Our study has several limitations. First, the cross-sectional design precludes the determination of temporal or causal relationships between seminal HDL-C depletion and impaired sperm quality. Second, although additional adjusted correlation analyses were performed to account for differences in serum FSH, estradiol, age, and smoking status, residual endocrine confounding cannot be fully excluded. In this respect, elevated estradiol in infertile men has been attributed to increased peripheral aromatization, and estrogens may modulate Sertoli-cell metabolic and lipid-regulatory processes, thereby potentially influencing the local seminal environment and complicating the interpretation of local lipid alterations [22,32]. Third, sperm concentration was assessed using a Makler counting chamber as part of the routine laboratory workflow, which may introduce some degree of measurement variability, particularly in samples with low sperm concentrations. Fourth, seminal lipid assessment was limited to quantitative measurements of total cholesterol and HDL-C, whereas subfraction analysis was performed only in serum. This was primarily because the Lipoprint HDL system, used for serum subfraction analysis in the present study, is a polyacrylamide gel electrophoresis-based platform validated exclusively for serum and plasma matrices. No standardized or commercially validated method currently exists for HDL subfraction quantification in seminal plasma, and the complex physicochemical properties of this matrix present additional analytical challenges. Seminal HDL subfraction profiling, therefore, remains an important avenue for future investigation. Fifth, the correlation analyses reported in the present study were performed across the full cohort. Because the two groups differ markedly in both seminal HDL-C levels and semen parameters, these associations may partly reflect between-group differences rather than within-group biological relationships. Subgroup analyses within each group were limited by reduced statistical power due to smaller sample sizes and restricted variance, and future larger studies are warranted to confirm these associations within homogeneous subgroups. Finally, although HDL particles are known to carry antioxidant-associated components and prior studies have linked paraoxonase family members and oxidative stress-related pathways to semen quality [26,28,33], we did not directly measure reactive oxygen species, oxidative damage markers, paraoxonase activity, total antioxidant capacity, or sperm DNA fragmentation. Accordingly, any mechanistic link between reduced seminal HDL-C and oxidative or genomic sperm injury remains hypothetical and should be examined in future studies incorporating functional biochemical and DNA integrity assays.

Overall, the present findings support a model in which lipid dysregulation in men with an OAT phenotype is more evident in the seminal compartment than in the systemic circulation. Preserved serum HDL subfraction profiles together with reduced seminal HDL-C suggest that local cholesterol homeostasis may be disrupted despite apparently normal systemic lipid status. This compartment-specific pattern reinforces the biological relevance of the seminal microenvironment in male infertility and identifies seminal HDL-C as a candidate local metabolic indicator deserving further investigation.

## 5. Conclusions

This study suggests a compartment-specific pattern of lipid alteration in men with an OAT phenotype, characterized by preserved serum HDL subfraction profiles but significantly reduced seminal HDL-C concentrations. The statistically significant weak-to-moderate positive associations between seminal HDL-C and key semen quality parameters suggest that local seminal lipid homeostasis may be more relevant to sperm functional competence than circulating HDL-related measures alone. These findings support seminal HDL-C as a candidate local metabolic indicator and further suggest that compartmentalized cholesterol dysregulation may contribute to the pathophysiology of male infertility.

## Figures and Tables

**Table 1:** Demographic and systemic metabolic characteristics of the study population.

Parameter	Normozoospermic (*n* = 50)	OAT (*n* = 49)	*p*-Value
Age (years)	35.5 (30–41)	35 (30–41)	0.916 *
Height (cm)	177 (172–182)	177 (172.5–182)	0.723 *
Weight (kg)	81.5 (72.7– 91.5)	82 (72.5–90.5)	0.93 *
BMI (kg/m^2^)	25.6 (23.6–30.0)	25.9 (23–29.4)	0.908 *
Smoking (*n*, %)	11 (22.0%)	11 (22.5%)	0.957 ^#^
Alcohol (*n*, %)	8 (16.0%)	9 (18.4%)	0.755 ^#^
Fasting Glucose (mg/dL)	92 (84–105.2)	93 (86–97.5)	0.997 *
Total Cholesterol (mg/dL)	207 (180–240)	205 (173–229)	0.416 *
Triglycerides (mg/dL)	131.5 (91.7–198)	135 (92.5–203)	0.997 *
HDL Cholesterol (mg/dL)	50.3 (45.75–56.8)	50.0 (45–54.8)	0.766 *
LDL Cholesterol (mg/dL)	125 (102–154.2)	123 (98.5–151.5)	0.607 *
Non-HDL Cholesterol (mg/dL)	159 (128.7–186)	149 (128–183.5)	0.607 *

Continuous variables are presented as Median (Q1–Q3) due to non-normal distribution. Categorical variables are presented as frequency (percentage). * Calculated using the Mann–Whitney U test. # Calculated using Pearson’s Chi-square test. BMI: Body Mass Index, HDL: High-Density Lipoprotein, LDL: Low-Density Lipoprotein.

**Table 2:** Endocrine profile and conventional semen parameters of the study population.

Parameter	Normozoospermic (*n* = 50)	OAT (*n* = 49)	*p*-Value
Follicle-Stimulating Hormone (mIU/mL)	3.76 (2.75–5.65)	5.20 (3.61–8.2)	0.008 *
Luteinizing Hormone (mIU/mL)	3.83 (2.53–4.8)	4.17 (3.24–5.35)	0.115 *
Total Testosterone (ng/dL)	357.6 (276.3–503.2)	437.5 (315.1–516.2)	0.173 *
Estradiol (pg/mL)	29.2 (24–35)	33.6 (29–38)	0.015 *
Semen Volume (mL)	3.5 (2.95–4.4)	3.6 (2.7–4.5)	0.974 *
Sperm Concentration (×10^6^/mL)	70 (48.7–88.2)	4.6 (1.7–8.5)	<0.001 *
Total Sperm Count (×10^6^/ejaculate)	250.6 (147.6–321.6)	14.1 (5.7–35.2)	<0.001 *
Total Motile Sperm Count (×10^6^/ejaculate)	115.5 (65.6–164.2)	4.36 (1.09–10.1)	<0.001 *
Rapid Progressive Motility (%)	15.5 (14–18)	9 (6–10)	<0.001 *
Slow Progressive Motility (%)	23 (20–26.25)	15 (12.5–18.5)	<0.001 *
Non-Progressive Motility (%)	8 (7–8)	6 (5–6.5)	<0.001 *
Immotile Sperm (%)	54 (46–59)	70 (66–76)	<0.001 *
Normal Morphology (%)	6 (5–6)	0 (0–1)	<0.001 *

Data are presented as Median (Q1–Q3) because the variables are non-normally distributed. * Calculated using the Mann–Whitney U test. OAT: Oligoasthenoteratozoospermia.

**Table 3:** Comparison of systemic serum HDL subfractions and localized seminal lipids.

Parameter	Normozoospermic (*n* = 50)	OAT (*n* = 49)	*p*-Value
Large HDL (mg/dL)	12 (9–15.25)	11 (9–15)	0.626
Intermediate HDL (mg/dL)	24 (22–27)	24 (21–26.5)	0.817
Small HDL (mg/dL)	13 (11–17)	13 (12–16)	0.852
HDL Subfraction 1 (mg/dL)	4 (2.75–6)	4 (2.50–6)	0.669
HDL Subfraction 2 (mg/dL)	4.5 (3–6)	4 (3–6)	0.907
HDL Subfraction 3 (mg/dL)	3 (3–4)	3 (2.50–5)	0.549
HDL Subfraction 4 (mg/dL)	5 (4–6)	5 (4–6)	0.692
HDL Subfraction 5 (mg/dL)	6 (5–7)	6 (5–6.5)	0.331
HDL Subfraction 6 (mg/dL)	10 (8.75–11)	10 (8–11)	0.664
HDL Subfraction 7 (mg/dL)	4 (3–4)	4 (3–4)	0.622
HDL Subfraction 8 (mg/dL)	3 (3–4)	4 (3–4)	0.798
HDL Subfraction 9 (mg/dL)	3 (3–4)	3 (2–3)	0.345
HDL Subfraction 10 (mg/dL)	7 (6–9)	7 (6–9)	0.832
Seminal total cholesterol (mg/dL)	20.6 (16.9–26.07)	18.5 (13.1–24.1)	0.048
Total seminal cholesterol content (mg/ejaculate)	0.74 (0.5–0.95)	0.62 (0.41–0.94)	0.194
Seminal HDL-C (mg/dL)	9.65 (7.71–12.4)	7.3 (5.81–9.36)	<0.001
Total seminal HDL-C content (mg/ejaculate)	0.31 (0.23–0.46)	0.24 (0.16–0.37)	0.01

Data are presented as Median (Q1–Q3) due to non-parametric distribution. Statistical comparisons between groups were performed using the Mann–Whitney U test. HDL: High-Density Lipoprotein, HDL-C: High-Density Lipoprotein-Cholesterol.

**Table 4:** Partial correlation analysis between localized seminal lipids and conventional semen parameters, controlling for serum FSH, estradiol, age, and smoking status.

Spermatological Parameters	Seminal Total Cholesterol		Seminal HDL Cholesterol	
	Partial *r*	*p*-Value	Partial *r*	*p*-Value
Rapid Progressive Motility (%)	0.333	<0.01	0.409	<0.01
Slow Progressive Motility (%)	0.381	<0.01	0.379	<0.01
Normal Morphology (%)	0.262	0.01	0.346	<0.01

The analysis was controlled for systemic Follicle-Stimulating Hormone (FSH), Estradiol (E2), age, and smoking status. Partial r represents the correlation coefficient after statistically eliminating the confounding effects of these variables. HDL: High-Density Lipoprotein.

## Data Availability

Data will be made available on request.
